# Clinical Details of Low-Frequency Hearing Loss Observed in Autosomal Dominant *MYO7A*-Associated Hearing Loss Patients

**DOI:** 10.3390/genes17030314

**Published:** 2026-03-11

**Authors:** Hiromi Koizumi, Shin-ya Nishio, Shin-ichi Usami

**Affiliations:** 1 Department of Otorhinolaryngology, Kyouai Hospital, Fujinomiya 418-0067, Japan; 2 Department of Hearing Implant Sciences, Shinshu University School of Medicine, Matsumoto 390-8621, Japan; nishio@shinshu-u.ac.jp

**Keywords:** *MYO7A*, autosomal dominant hearing loss, DFNA11, low-frequency hearing loss, next-generation sequencing

## Abstract

Background/Objectives: *MYO7A* is known to be the genetic cause of Usher syndrome type 1, as well as autosomal dominant and autosomal recessive non-syndromic hearing loss. In general, autosomal dominant *MYO7A*-associated hearing loss shows progressive high-frequency, sloping hearing loss. However, several variants are associated with low-frequency hearing loss. *MYO7A*-associated low-frequency hearing loss is relatively rare, and the clinical details remain unclear. Methods: A total of 18,475 Japanese patients with hearing loss were recruited. Targeted massively parallel sequencing of 158 deafness-related genes was performed, and individuals with variants related to *MYO7A*-associated low-frequency hearing loss were identified. Results: Among 18,475 hearing loss patients, we identified 60 patients from 44 unrelated families carrying five variants (p.[Asn140Lys; Glu1835Gln], p.Leu479Pro, p.Leu656Val, p.Gly660Arg, and p.Arg668His) for *MYO7A*-associated low-frequency hearing loss. Patients identified in this study initially showed postlingual-onset mild-to-moderate low-frequency hearing loss; however, high-frequency hearing also deteriorated after the fourth decade, eventually leading to moderate-to-severe flat-type hearing loss. In addition, we performed haplotype analysis for the recurrent variant c.1436T>C:p.Leu479Pro identified in this study and found that this variant is a founder mutation in the Japanese population. Conclusions: In this study, we were able to clarify the specific features of *MYO7A*-related low-frequency hearing loss in a significant number of patients. In particular, we clarified the details of hearing deterioration at each frequency. Our findings will be useful for providing more appropriate treatment and follow-up for *MYO7A*-associated low-frequency hearing loss.

## 1. Introduction

Hearing loss (HL) is one of the most common sensory disorders, with more than 150 genes identified to date as causative of non-syndromic HL [1]. *MYO7A* is a frequent genetic cause of Usher syndrome type 1 (USH1B), which causes congenital severe-to-profound bilateral sensorineural HL (SNHL), prepubertal-onset retinitis pigmentosa (PR), and vestibular dysfunction [2]. In addition, *MYO7A* is known as a causative gene for autosomal dominant non-syndromic HL (ADNSHL, locus DFNA11) and autosomal recessive non-syndromic HL (ARNSHL, locus DFNB2) [3,4].

The *MYO7A* gene, initially identified as a causative gene for Usher syndrome by Weil et al. in 1995 [1], is located on chromosome 11q13.5, comprises 49 exons, and encodes the unconventional myosin VIIa. Myosin VIIa is expressed in multiple tissues, including the retina, lungs, testes, kidneys, and both the outer and inner hair cells of the inner ear [4]. Within the inner ear, myosin VIIa interacts with SANS and harmonin to form a tripartite protein complex that is critical for mechanoelectrical transduction in stereocilia [5,6,7,8,9,10]. It also plays a pivotal role in maintaining mechanical tension through tip links and facilitating the transport of protein components to the tips of stereocilia [7,10]. The integrity of hair cell stereocilia bundles depends on myosin VIIa function, and its disruption leads to disorganized stereocilia and subsequent HL [11,12].

To date, a total of 882 variants have been reported in association with *MYO7A*-related HL and Usher syndrome. The majority of these variants have been linked to Usher syndrome, whereas only 35 have been identified as causative of ADNSHL and 49 as causative of ARNSHL [13]. We recently described the clinical characteristics of *MYO7A*-related HL, including DFNA11, DFNB2, and USH1B [14]. In that study, DFNA11 cases typically showed late-onset progressive SNHL, with onset ranging from the first to the third decade. Most DFNA11 patients initially presented with mild-to-moderate high-frequency sloping SNHL, gradually worsening to a severe-to-profound flat-type configuration with age [14]. DFNB2 cases exhibited congenital or early-onset progressive SNHL. Although the age of onset was consistently within the first decade, severity and audiometric patterns varied across individuals. Around half of those over 30 years of age showed severe-to-profound SNHL, confirming a progressive course [14]. In contrast, USH1B cases uniformly demonstrated congenital or early onset severe-to-profound SNHL. All individuals over 10 years old developed retinitis pigmentosa and/or related visual symptoms [14].

As described above, most DFNA11 cases in our previous study showed high-frequency sloping SNHL. Xia et al. reported the results of a literature review on DFNA11 and found that six of 11 families with DFNA11 showed high-frequency sloping SNHL [15]. However, several patients with specific variants showed low-frequency SNHL. Low-frequency HL is a relatively rare phenotype for DFNA11, and the clinical details, including the patterns of hearing deterioration, remain unclear. We, therefore, collected patients with specific *MYO7A* variants consistently associated with a low-frequency HL pattern to clarify the clinical details. For this purpose, we analyzed the detailed clinical features (including the audiometric configuration and hearing deterioration at each frequency by age) of DFNA11 cases with low-frequency HL. We also performed haplotype analysis for a recurrent variant (c.1436T>C:p.Leu479Pro) identified in 29 independent families among 18,475 Japanese HL patients.

## 2. Materials and Methods

### 2.1. Subjects

A total of 18,475 HL patients from 130 institutions in Japan participated in this study. Clinical information and peripheral blood samples were obtained from each participant. Written informed consent was obtained from all participants (or guardians in the case of minors) prior to participation in the project. This study was performed in accordance with the Declaration of Helsinki. The study protocol was approved by the Shinshu University School of Medicine Ethics Committee (No. 387—4 September 2012, No. 576—2 May 2017, and No. 718—7 March 2022).

### 2.2. Next-Generation Sequencing and Bioinformatic Analysis

Next-generation sequencing (NGS) analysis for 158 target genes reported to cause non-syndromic HL or syndromic HL was performed. In brief, sequencing libraries were prepared with an Ion AmpliSeq Library Kit 2.0 (ThermoFisher Scientific, Waltham, MA, USA) and Ion AmpliSeq^TM^ Custom Panel (ThermoFisher Scientific) according to the manufacturer’s procedure [16]. Sequencing was performed using the Ion S5 Plus system with the Ion 540 Chip Kit and Ion 540 Kit-Chef (ThermoFisher Scientific). The sequence data were mapped against the human genome sequence (build GRCh37/hg19), and variants were picked up with the Torrent Suite software ver.5.1.6. The annotation of identified variants was performed with ANNOVAR software ver. 2020-06-08 [17]. Variants affecting the amino acid sequence (missense, nonsense, splicing, and insertion/deletion variants) were selected from the identified variants. Variants were further restricted to those with a minor allele frequency of less than 1% in control databases. As control databases, we employed the Genome Aggregation Database (https://gnomad.broadinstitute.org), ToMMo 60KJPN (https://jmorp.megabank.tohoku.ac.jp/, accessed on 10 October 2024), and 333 in-house normal-hearing Japanese controls. All variant filtering was performed with our original database software [18]. Copy number analysis was performed according to our previous report [19]. Sanger sequencing was conducted to confirm the identified variants and family segregation.

The pathogenicity of the selected variants was assessed in accordance with the American College of Medical Genetics (ACMG) standards and guidelines [20] with the ClinGen Hearing Loss Clinical Domain Working Group expert specification [21]. The variants classified as “Likely Pathogenic” or “Pathogenic” were considered to be causative variants. In addition, variants classified as being of “Uncertain Significance” were considered to be causative variants if all of the following conditions were fulfilled: (1) no other candidate variants were identified among the other 157 genes, (2) the allele frequency was extremely low (≤0.00002) in the control populations, (3) most of the in silico prediction scores supported the pathogenic impact, and (4) no contradictory evidence existed regarding the pathogenicity of the identified variant.

### 2.3. Clinical Evaluation

Clinical data, including sex, age at onset of HL, age and audiometric thresholds at the time of genetic testing, family history, history of tinnitus and vertigo, and hearing loss progression, were obtained through a retrospective review of medical records. Hearing thresholds were assessed using pure-tone audiometry in individuals aged over 5 years. Conditioned orientation reflex audiometry (COR), play audiometry or auditory steady-state response (ASSR) was performed in younger children. The pure-tone average (PTA) was calculated based on the hearing thresholds at four frequencies (500, 1000, 2000, and 4000 Hz). Severity of HL was categorized according to PTA values: normal (<25 dB), mild (>25 dB and ≤40 dB HL), moderate (>40 dB and ≤70 dB HL), severe (>70 dB and ≤90 dB HL), and profound (>90 dB HL). Type of HL were classified into flat, low-frequency ascending, mid-frequency U-shaped, high-frequency gently sloping, and high-frequency steeply sloping [22].

### 2.4. Haplotype Analysis

In this study, we identified a recurrent variant, c.1436T>C, from 29 independent families. To determine whether this variant resulted from a mutational hotspot or founder mutation, we performed haplotype analysis. The haplotype pattern within the 1 Mbp region surrounding the position of the most frequent variant, c.1436T>C, was characterized using a set of 23 tag single-nucleotide polymorphisms (SNPs). Tag SNPs were selected based on the minor allele frequency for the 1000 Genomes JPT populations using SNPinfo Web Server LD TAG SNP Selection (TagSNP) (https://snpinfo.niehs.nih.gov). SNP genotyping for haplotype analysis was performed by Sanger sequencing.

## 3. Results

### 3.1. Identified Variants and Clinical Features of MYO7A-Associated Low Frequency Hearing Loss

In this study, we evaluated the audiogram for 60 cases, including those from our previous study [14], and found that the five variants, p.[Asn140Lys; Glu1835Gln], p.Leu479Pro, p.Leu656Val, p.Gly660Arg, and p.Arg668His, were associated with low-frequency HL.

To elucidate the detailed clinical features of *MYO7A*-associated low-frequency HL, we selected patients carrying the above-mentioned five variants from the 18,475 HL patient cohort. As a result, we identified 60 patients from 44 unrelated families carrying these five variants associated with low-frequency HL (Table 1). The most prevalent variant was p.Leu479Pro, which was identified in 40 cases from 28 families, followed by p.Leu656Val in nine cases from nine families, p.Arg668His in five cases from three families, p.Gly660Arg in four cases from two families, and p.[Asn140Lys; Glu1835Gln] in two cases from two families. Twenty-two patients were male, and 38 were female. Most of the cases (84.1%, 37/44) were from families with an autosomal dominant family history, which was defined as one or more affected individuals among first-degree relatives. The average onset age of HL for these patients was 28.5 years (range 0 to 64 years old). Most cases (91.7%, 44/48) showed postlingual-onset HL, and only four cases (8.3%, 4/48) showed prelingual-onset HL. Most of the identified variants were only observed in Japanese patients and were not observed in a large-scale control database (gnomAD ver 4.1). On the other hand, p.Arg668His was also observed in Chinese and American patients (Table 2).

Among the 60 patients, audiometric data were available for 57 patients. Most cases showed mild-to-moderate HL: 20 patients showed mild HL (35.1%, 20/57), 29 patients showed moderate HL (51.0%, 29/57), five patients showed severe HL (8.8%, 5/57), and one patient showed profound HL. In addition, two cases showed normal hearing on PTA at the time of genetic testing. Of these 57 patients, 34 patients (59.6%) exhibited low-frequency HL, whereas 21 patients (36.8%) showed flat-type HL, and one patient each (1.8%) exhibited high-frequency HL and mid-frequency HL. Progressive HL and tinnitus were the major subjective symptoms: 70.8% of patients (34/48) were aware of their HL progression, and 61.8% of patients (34/55) had tinnitus. On the other hand, vertigo was a relatively minor symptom, with 28.6% of patients (16/56) reporting episodes of vertigo. As a limitation of this study, information on the progression of HL, tinnitus, and vertigo was based on subjective awareness, and longitudinal objective measurements would be useful to clarify the true prevalence of each symptom.

**Figure 1:**
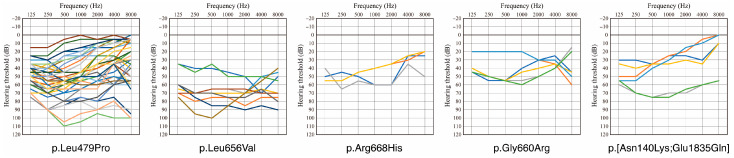
Overlapping audiograms of the patients with *MYO7A*-associated HL for each variant. This figure was prepared using the hearing thresholds for the better-hearing ear. Different colors indicate the hearing thresholds for different individuals.

Overlapping audiograms of the patients identified in this study for each variant are shown in Figure 1. As shown in Figure 1, most individuals with mild-to-moderate HL showed a typical low-frequency HL phenotype. On the other hand, many patients with moderate-to-severe HL showed flat-type HL. Thus, we hypothesized that *MYO7A*-associated HL caused by these five variants initially presents as mild-to-moderate low-frequency HL that progresses to moderate-to-severe flat-type HL.

### 3.2. Hearing Deterioration in MYO7A-Associated Low-Frequency Hearing Loss

**Figure 2:**
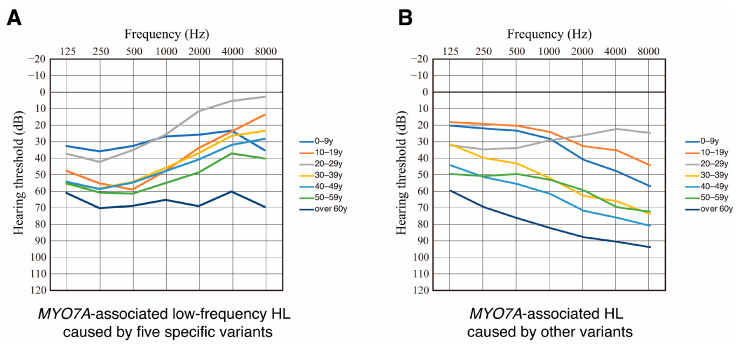
Mean hearing thresholds for patients with *MYO7A*-associated HL at each decade of age. (**A**) Mean hearing thresholds for patients with the five specific variants at each decade of age. (**B**) Mean hearing thresholds for the patients with other variants at each decade of age.

To elucidate hearing deterioration in patients with *MYO7A*-associated low-frequency HL, we evaluated the averaged hearing thresholds for each decade of age group for patients with the five specific *MYO7A* variants (Figure 2A). To highlight the differences in hearing deterioration between *MYO7A*-associated low-frequency HL and other DFNA11 cases, we also evaluated the averaged hearing thresholds for each decade of age group in DFNA11 patients with other *MYO7A* variants (Figure 2B). As seen in Figure 2, patients with the five specific variants showed clear low-frequency HL from the second to the sixth decade, with HL later deteriorating at higher frequencies, resulting in flat-type HL. On the other hand, patients carrying other DFNA11 variants showed high-frequency sloping HL that deteriorated across all frequencies.

**Figure 3:**
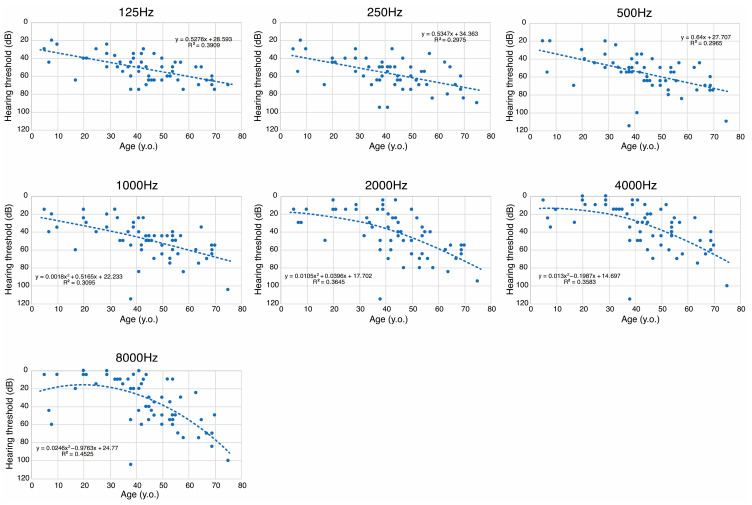
Scatter plots and regression analysis for patients with *MYO7A*-associated low-frequency HL identified in this study. Hearing thresholds of the better-hearing ear at 125, 250, 500, 1000, 2000, 4000, and 8000 Hz, along with age at hearing testing, were plotted. The dashed line indicates the linear or quadratic regression curve. Linear regression was applied for 125, 250, and 500 Hz, and quadratic regression was applied for 1000, 2000, 4000, and 8000 Hz.

We also performed a scatter plot analysis of hearing thresholds at each frequency (125 Hz, 250 Hz, 500 Hz, 1000Hz, 2000 Hz, 4000 Hz, and 8000 Hz) by age at audiometric testing (Figure 3). As shown in Figure 3, hearing levels at low frequencies showed progressive deterioration beginning in the first or second decade (>25 dB), with a linear decline at a rate of 0.5–0.6 dB/year. In contrast, hearing levels at higher frequencies remained within the normal range (<25 dB) from the first to the third decade. However, HL beginning in the fourth decade showed more rapid deterioration at higher frequencies than at lower frequencies. Thus, based on the scatter plot analysis results, we clearly demonstrated that patients with *MYO7A*-associated low-frequency HL initially presented with mild-to-moderate low-frequency HL that progressed with time to moderate-to-severe flat-type HL. It is noteworthy that there was a large variability in the individual progression of HL, and scatter plot analysis showed overall trends for hearing deterioration in *MYO7A*-associated low-frequency HL.

### 3.3. Haplotype Analysis for the Recurrent MYO7A Variant Identified in This Study

**Figure 4:**
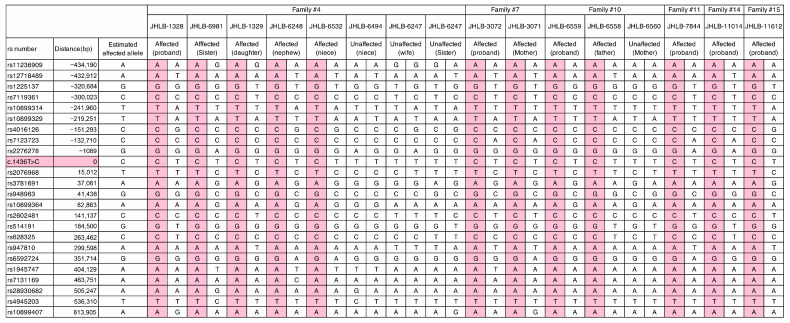
Haplotype analysis for 16 individuals from 6 independent families within the 1 Mbp region surrounding the position of the c.1436T>C variant. Pink indicates the common haplotype observed in affected individuals.

In this study, we identified 38 cases from 29 unrelated families carrying the same c.1436T>C:p.Leu479Pro variant. It is possible that this recurrent variant may be due to a mutational hotspot or founder mutation. To elucidate this possibility, we carried out haplotype analysis in 16 individuals from six independent families within the 1 Mbp region surrounding the position of c.1436T>C, which was characterized using a set of 23 single-nucleotide polymorphisms (SNPs) (9 sites upstream and 14 sites downstream). Figure 4 shows the haplotype patterns for the six families that carried the c.1436T>C variant. As a result, the six unrelated families were found to share the same haplotype surrounding the c.1436T>C variant, suggesting that this mutation likely occurred and spread as a founder mutation in the Japanese population.

## 4. Discussion

*MYO7A* is known to be the genetic cause of Usher syndrome type 1 (USH1B), ADNSHL (DFNA11), and ARNSHL (DFNB2) [3,4]. Most of the previously reported variants were identified from Usher syndrome patients, while DFNA11 cases were relatively rare, and details of the clinical characteristics remain unclear. DFNA11 HL is generally characterized by a high-frequency sloping audiometric configuration with progressive HL [14,23]. However, several variants have been associated with low-frequency HL.

In this study, we analyzed the detailed clinical features, especially hearing deterioration, in DFNA11 patients with low-frequency HL caused by five specific variants (p.Leu479Pro, p.Leu656Val, p.Gly660Arg, p.Arg668His, and p.[Asn140Lys; Glu1835Gln]). As a result, DFNA11 patients with these five specific variants generally presented with postlingual-onset mild-to-moderate low-frequency HL. Hearing thresholds for 125, 250, and 500 Hz deteriorated at a rate of 0.5 to 0.6 dB/year in a linear manner (Figure 3). On the other hand, hearing thresholds at 2000, 4000, and 8000 Hz were within the normal range in the first to third decades, but rapidly deteriorated after the fourth decade and finally progressed to moderate-to-severe flat-type HL (Figure 2 and Figure 3). These estimates of hearing deterioration were based on mean thresholds by decade (Figure 2), scatter plots of different patients (Figure 3), and an analysis of hearing deterioration analysis based on longitudinal observations of the same individuals will be required to clarify the variability between patients carrying each variant.

The encoded protein myosin VIIa is localized in the stereocilia of the inner and outer hair cells and plays a crucial role in sound transduction, but the underlying mechanism for different types of HL (five variants resulting in low-frequency HL, whereas other DFNA11 variants generally cause high-frequency HL) remains unclear. The myosin VIIa protein is composed of a myosin motor domain (65–741 aa), a neck domain with five IQ motifs (745–857 aa), an SAH plus short coiled-coil domain (858–935 aa), and a tail domain that contains the MyTH4 1 domain (1017–1253 aa), FERM 1 domain (1258–1602 aa), SH3 domain (1603–1672 aa), MyTH4 2 domain (1747–1896 aa), and FERM 2 domain (1902–2205 aa) [24]. In a previous report, most *MYO7A* variants causing DFNA11 or DFNB2 were found to be located in the motor domain, while variants responsible for USH1B were observed across all domains [25].

Among the five variants identified in this study, p.Leu479Pro, p.Leu656Val, p.Gly660Arg, and p.Arg668His are located in the motor domain. In a recent review paper, five different variants, p.Arg616Gln, which was identified from Korean patients; Arg668His, which was identified from Chinese, Japanese, and American patients; Gly671Ser, which was identified from Chinese patients; p.Gly722Arg, which was identified from American patients; and p.Arg853Cys, which was identified from German patients, were reported to be causative of DFNA11 with low-frequency HL [26]. Among them, p.Arg616Gln, Arg668His, Gly671Ser, and p.Gly722Arg are also located in the motor domain. The motor domain can be divided into the N-terminal subdomain, Upper 50 kDa subdomain, Lower 50 kDa subdomain, and converter/lever-arm region. Among the above- mentioned variants identified in this study and previous reports, p.Leu479Pro and p.Arg616Gln are localized in the Lower 50 kDa subdomain, whereas p.Leu656Val, p.Gly660Arg, p.Arg668His, Gly671Ser, and p.Gly722Arg are localized in the converter/lever-arm region. Thus, there might be a genotype-phenotype correlation between the variant localization and type of HL; however, the detailed mechanisms remain unclear. Kallman et al. reported an interesting DFNA11 family in which some patients in a specific branch of the large family showed high-frequency HL, whereas other patients showed low-frequency HL despite carrying the same *MYO7A* variant. They speculated that a modifier gene may be involved in these phenotypic differences; however, this modifier gene remains unclear [27]. Further studies, including the prediction of protein structure changes, will be needed to elucidate the mechanisms for the different phenotypes observed in DFNA11 patients.

Among cases of hereditary HL, low-frequency HL is known to be classically associated with genes such as the *WFS1* gene [28]. In a recent review article on the monogenic causes for low-frequency non-syndromic HL, most cases of *WFS1*-associated HL (DFNA6/14/38), and some cases of *DIAPH1*-associated HL (DFNA1), *MYO7A*-associated HL (DFNA11), *TNC*-associated HL (DFNA56), and *CCDC50*-associated HL (DFNA44) showed low-frequency HL [26]. Based on our study, when evaluating patients with low-frequency HL, *MYO7A*-related HL should also be considered in the interpretation of next-generation sequencing results.

In this study, we identified 38 cases from 29 unrelated families carrying the same c.1436T>C:p.Leu479Pro variant. Recurrent pathogenic variants are generally considered to arise through two principal mechanisms: a founder effect or the presence of mutational hot spots. To distinguish between these mechanisms, haplotype analysis is a widely used and effective approach. To date, several recurrent variants have been shown to result from founder effects, including variants in *CDH23* [29], *MYO15A* [30], and *TMC1* [31]. In Japanese patients, founder mutations have also been reported in multiple genes. Haplotype analyses have suggested that p.Arg1939Gln in *OTOF* [32], c.211delC in *KCNQ4* [33], and c.4212+1G>A in *LOXHD1* [34] are likely founder variants. In contrast, recurrent variants such as c.5597C>T in *TECTA* [35] and p.Ala716Thr, p.Lys836Thr, and p.Glu864Lys in *WFS1* [28] have been attributed to mutational hot spots. In the present study, we investigated the high prevalence of the *MYO7A* p.Leu479Pro variant in the Japanese population to determine whether its recurrence is attributable to a founder effect or to a mutational hot spot. Haplotype analysis revealed a shared haplotype among affected individuals from unrelated families, indicating that this variant represents a founder mutation. Further, the p.Leu479Pro variant was not observed in the gnomAD database [36] and has only reported from the Japanese population, which indicates that this variant likely first occurred in a Japanese ancestor. Interestingly, all of the patients with this variant were living on the main island of Japan (Honshu), and no patients were observed on the northern island (Hokkaido) or the southern island (Kyushu), which also supports the idea that this variant arose as a founder mutation and spread from a common ancestor.

## 5. Conclusions

In this study, we were able to clarify the detailed characteristics of HL for *MYO7A*-associated low-frequency HL in a significant number of patients. The most notable result of this study was our ability to clarify the details of hearing deterioration at each frequency. Hearing thresholds for 125, 250, and 500 Hz deteriorated at a rate of 0.5 to 0.6 dB/year in a linear manner. On the other hand, hearing thresholds for 2000, 4000, and 8000 Hz rapidly deteriorated after the fourth decade and progressed to moderate-to-severe flat-type HL. In addition, we performed haplotype analysis for the recurrent c.1436T>C:p.Leu479Pro variant identified in this study and determined that this variant was likely derived from a founder mutation that occurred in a common ancestor. The findings of this study will be beneficial in enabling more appropriate treatment for patients with *MYO7A*-associated low-frequency HL based on the expectation of future hearing deterioration.

## Figures and Tables

**Table 1:** Patients possessing the five variants causative for *MYO7A*-associated low-frequency HL.

Family No.	ID	Gender	Inheritance	Base Change	AA Change	Age	Onset of HL	Type of HL	Severity	Progression	Tinnitus	Vertigo
1	JHLB-7873	M	AD	c.[420C>G;5503G>C]	p.[Asn140Lys;Glu1835Gln]	43	38	flat	mild	no	yes	no
2	JHLB-9789	M	AD	c.[420C>G;5503G>C]	p.[Asn140Lys;Glu1835Gln]	41	32	LF	mild	yes	no	no
3	JHLB-0324	M	AD	c.1436T>C	p.Leu479Pro	47	N/A	LF	moderate	yes	yes	no
4	JHLB-1328	M	AD	c.1436T>C	p.Leu479Pro	45	0	flat	moderate	no	no	no
	sister	F		c.1436T>C	p.Leu479Pro	54	N/A	flat	moderate	yes	yes	no
	daughter	F		c.1436T>C	p.Leu479Pro	20	0	LF	mild	no	no	no
	nephew	M		c.1436T>C	p.Leu479Pro	19	N/A	LF	normal	no	no	no
	niece	F		c.1436T>C	p.Leu479Pro	29	29	LF	mild	no	no	yes
5	JHLB-1388	F	AD	c.1436T>C	p.Leu479Pro	38	15	flat	moderate	yes	no	no
6	JHLB-2474	F	sporadic	c.1436T>C	p.Leu479Pro	42	31	flat	moderate	yes	no	no
7	JHLB-3072	F	AD	c.1436T>C	p.Leu479Pro	21	13	LF	mild	no	no	no
	mother	F		c.1436T>C	p.Leu479Pro	50	13	LF	moderate	yes	yes	no
8	JHLB-4697	M	AD	c.1436T>C	p.Leu479Pro	53	52	MF	severe	yes	yes	no
9	JHLB-4824	M	AD	c.1436T>C	p.Leu479Pro	53	30	flat	moderate	yes	yes	yes
10	JHLB-6559	F	AD	c.1436T>C	p.Leu479Pro	5	3	LF	normal	N/A	NA	no
	father	M		c.1436T>C	p.Leu479Pro	44	30	LF	moderate	yes	no	no
11	JHLB-7844	M	AD	c.1436T>C	p.Leu479Pro	55	47	flat	moderate	yes	yes	yes
12	JHLB-10054	F	AD	c.1436T>C	p.Leu479Pro	57	55	flat	moderate	yes	no	no
13	JHLB-10055	F	AD	c.1436T>C	p.Leu479Pro	33	N/A	LF	mild	yes	no	no
	sister	F		c.1436T>C	p.Leu479Pro	37	32	LF	mild	yes	yes	yes
14	JHLB-11014	F	AD	c.1436T>C	p.Leu479Pro	35	25	LF	mild	yes	yes	yes
15	JHLB-11612	M	AD	c.1436T>C	p.Leu479Pro	70	40	LF	moderate	yes	yes	no
16	JHLB-13274	F	AD	c.1436T>C	p.Leu479Pro	38	N/A	flat	profound	no	yes	no
17	JHLB-14272	M	sporadic	c.1436T>C	p.Leu479Pro	34	32	LF	mild	N/A	yes	yes
18	JHLB-14286	F	AD	c.1436T>C	p.Leu479Pro	69	40	flat	moderate	yes	yes	yes
19	JHLB-14914	F	sporadic	c.1436T>C	p.Leu479Pro	45	20	LF	moderate	yes	yes	no
20	JHLB-14931	M	AD	c.1436T>C	p.Leu479Pro	29	N/A	LF	mild	NA	yes	yes
21	JHLB-15583	F	AD	c.1436T>C	p.Leu479Pro	20	20	LF	mild	no	yes	no
	aunt	F		c.1436T>C	p.Leu479Pro	39	25	LF	mild	yes	no	no
	mother	F		c.1436T>C	p.Leu479Pro	44	28	N/A	N/A	no	yes	yes
22	JHLB-16124	F	AD	c.1436T>C	p.Leu479Pro	44	40	LF	mild	yes	no	no
	JHLB-16123	F		c.1436T>C	p.Leu479Pro	17	6	LF	moderate	no	no	no
23	JHLB-16263	F	AD	c.1436T>C	p.Leu479Pro	35	25	LF	mild	yes	yes	yes
24	JHLB-16419	F	AD	c.1436T>C	p.Leu479Pro	52	40	LF	mild	yes	yes	no
25	JHLB-17237	F	sporadic	c.1436T>C	p.Leu479Pro	54	45	flat	moderate	yes	no	no
26	HL12161	F	AD	c.1436T>C	p.Leu479Pro	39	33	LF	mild	NA	no	no
27	HL12686	F	AD	c.1436T>C	p.Leu479Pro	65	30	flat	moderate	yes	yes	yes
28	OHL-2341	M	AD	c.1436T>C	p.Leu479Pro	10	6	LF	mild	NA	yes	yes
	mother	F		c.1436T>C	p.Leu479Pro	N/A	25	LF	moderate	yes	yes	no
29	OHL-2963	M	AD	c.1436T>C	p.Leu479Pro	37	10	LF	moderate	yes	yes	no
	father	M		c.1436T>C	p.Leu479Pro	71	N/A	flat	severe	NA	N/A	N/A
30	OHL-3506	F	AD	c.1436T>C	p.Leu479Pro	75	55	flat	severe	yes	yes	no
31	JHLB-9793	M	sporadic	c.1966C>G	p.Leu656Val	41	10	LF	severe	yes	no	no
32	JHLB-12432	F	AD	c.1966C>G	p.Leu656Val	54	30	flat	moderate	yes	yes	no
33	JHLB-15696	M	AD	c.1966C>G	p.Leu656Val	69	60	N/A	N/A	yes	yes	yes
34	JHLB-16258	F	AD	c.1966C>G	p.Leu656Val	39	33	LF	mild	yes	no	no
35	HL16548	F	AD	c.1966C>G	p.Leu656Val	54	40	LF	moderate	no	yes	no
36	OHL171	M	AD	c.1966C>G	p.Leu656Val	N/A	42	flat	moderate	yes	yes	no
37	OHL-700	F	unknown	c.1966C>G	p.Leu656Val	N/A	64	flat	severe	NA	yes	no
38	OHL-778	M	AD	c.1966C>G	p.Leu656Val	N/A	40	flat	moderate	NA	yes	yes
39	OHL-1718	F	AD	c.1966C>G	p.Leu656Val	N/A	20	flat	moderate	no	yes	no
40	JHLB-5274	M	unknown	c.2003G>A	p.Arg668His	63	N/A	LF	moderate	no	no	no
41	JHLB-7703	F	AD	c.2003G>A	p.Arg668His	41	25	LF	moderate	yes	yes	yes
	mother	F		c.2003G>A	p.Arg668His	N/A	N/A	LF	moderate	N/A	N/A	N/A
	brother	M		c.2003G>A	p.Arg668His	N/A	N/A	flat	moderate	N/A	N/A	N/A
42	JHLB-14485	F	AD	c.2003G>A	p.Arg668His	29	28	LF	mild	yes	yes	yes
43	JHLB-0673	F	AD	c.1978G>A	p.Gly660Arg	7	N/A	LF	moderate	no	no	no
	mother	F		c.1978G>A	p.Gly660Arg	N/A	N/A	N/A	N/A	N/A	N/A	N/A
44	JHLB-1258	M	AD	c.1978G>A	p.Gly660Arg	42	4	flat	moderate	yes	yes	no
	daughter	F		c.1978G>A	p.Gly660Arg	8	7	HF	mild	N/A	no	no

M: Male, F: Female, AD: Autosomal dominant, LF: Low-frequency hearing loss, MF: Mid-frequency hearing loss, HF: High-frequency hearing loss, N/A: Data not available.

**Table 2:** Minor allele frequencies for the five variants causative for *MYO7A*-associated low-frequency HL in the gnomAD v4.1 database.

Base Change	AA Change	gnomAD all	gnomAD afr	gnomAD amr	gnomAD asj	gnomAD eas	gnomAD fin	gnomAD nfe	gnomAD sas	gnomAD oth	Ethnicity of Previously Reported Patients
c.[420C>G;5503G>C]	p.[Asn140Lys;Glu1835Gln]										Japanese
c.1436T>C	p.Leu479Pro										Japanese
c.1966C>G	p.Leu656Val										Japanese
c.1978G>A	p.Gly660Arg										Japanese
c.2003G>A	p.Arg668His	1.37 × 10^−6^						1.80 × 10^−6^			Chinese American Japanese

## Data Availability

The datasets used during the current study are available from the corresponding author on reasonable request.

## References

[1] Weil D., Blanchard S., Kaplan J., Guilford P., Gibson F., Walsh J., Mburu P., Varela A., Levilliers J., Weston M.D. (1995). Defective myosin VIIA gene responsible for Usher syndrome type 1B. Nature.

[2] Liu X.Z., Walsh J., Tamagawa Y., Kitamura K., Nishizawa M., Steel K.P., Brown S.D. (1997). Autosomal dominant non-syndromic deafness caused by a mutation in the myosin VIIA gene. Nat. Genet..

[3] Liu X.Z., Walsh J., Mburu P., Kendrick-Jones J., Cope M.J., Steel K.P. (1997). Mutations in the myosin VIIA gene cause non-syndromic recessive deafness. Nat. Genet..

[4] Hasson T., Heintzelman M.B., Santos-Sacchi J., Corey D.P., Mooseker M.S. (1995). Expression in cochlea and retina of myosin VIIa, the gene product defective in Usher syndrome type 1B. Proc. Natl. Acad. Sci. USA.

[5] Boëda B., El-Amraoui A., Bahloul A., Goodyear R., Daviet L., Blanchard S., Perfettini I., Fath K.R., Shorte S., Reiners J. (2002). Myosin VIIa, harmonin and cadherin 23, three Usher I gene products that cooperate to shape the sensory hair cell bundle. EMBO J..

[6] Bahloul A., Michel V., Hardelin J.P., Nouaille S., Hoos S., Houdusse A., England P., Petit C. (2010). Cadherin-23, myosin VIIa and harmonin, encoded by Usher syndrome type I genes, form a ternary complex and interact with membrane phospholipids. Hum. Mol. Genet..

[7] Grati M., Kachar B. (2011). Myosin VIIa and sans localization at stereocilia upper tip-link density implicates these Usher syndrome proteins in mechanotransduction. Proc. Natl. Acad. Sci. USA.

[8] Wu L., Pan L., Zhang C., Zhang M. (2012). Large protein assemblies formed by multivalent interactions between cadherin23 and harmonin suggest a stable anchorage structure at the tip link of stereocilia. J. Biol. Chem..

[9] He Y., Li J., Zhang M. (2019). Myosin VII, USH1C, and ANKS4B or USH1G Together Form Condensed Molecular Assembly via Liquid-Liquid Phase Separation. Cell Rep..

[10] Yu I.M., Planelles-Herrero V.J., Sourigues Y., Moussaoui D., Sirkia H., Kikuti C., Stroebel D., Titus M.A., Houdusse A. (2017). Myosin 7 and its adaptors link cadherins to actin. Nat. Commun..

[11] El-Amraoui A., Petit C. (2005). Usher I syndrome: Unravelling the mechanisms that underlie the cohesion of the growing hair bundle in inner ear sensory cells. J. Cell Sci..

[12] Underhill A., Webb S., Grandi F.C., Jeng J.Y., de Monvel J.B., Plion B., Carlton A.J., Amariutei A.E., Voulgari N., De Faveri F. (2025). *MYO7A* is required for the functional integrity of the mechanoelectrical transduction complex in hair cells of the adult cochlea. Proc. Natl. Acad. Sci. USA.

[13] Stenson P.D., Mort M., Ball E.V., Shaw K., Phillips A., Cooper D.N. (2014). The Human Gene Mutation Database: Building a comprehensive mutation repository for clinical and molecular genetics, diagnostic testing and personalized genomic medicine. Hum. Genet..

[14] Watanabe K., Nishio S.Y., Usami S.I., Deafness Gene Study Consortium (2024). The prevalence and clinical features of *MYO7A*-related hearing loss including DFNA11, DFNB2 and USH1B. Sci. Rep..

[15] Xia C.F., Yan R., Su W.W., Liu Y.H. (2023). Autosomal dominant non-syndromic hearing loss caused by a novel mutation in MYO7A: A case report and review of the literature. World J. Clin. Cases.

[16] Nishio S.Y., Hayashi Y., Watanabe M., Usami S. (2015). Clinical application of a custom AmpliSeq library and ion torrent PGM sequencing to comprehensive mutation screening for deafness genes. Genet. Test. Mol. Biomarkers.

[17] Wang K., Li M., Hakonarson H. (2010). ANNOVAR: Functional annotation of genetic variants from high-throughput sequencing data. Nucleic Acids Res..

[18] Nishio S.Y., Usami S.I. (2017). The Clinical Next-Generation Sequencing Database: A Tool for the Unified Management of Clinical Information and Genetic Variants to Accelerate Variant Pathogenicity Classification. Hum. Mutat..

[19] Nishio S.Y., Moteki H., Usami S.I. (2018). Simple and efficient germline copy number variant visualization method for the Ion AmpliSeq custom panel. Mol. Genet. Genom. Med..

[20] Richards S., Aziz N., Bale S., Bick D., Das S., Gastier-Foster J., Grody W.W., Hegde M., Lyon E., Spector E. (2015). Standards and guidelines for the interpretation of sequence variants: A joint consensus recommendation of the American College of Medical Genetics and Genomics and the Association for Molecular Pathology. Genet. Med..

[21] Oza A.M., DiStefano M.T., Hemphill S.E., Cushman B.J., Grant A.R., Siegert R.K., Shen J., Chapin A., Boczek N.J., Schimmenti L.A. (2018). Expert specification of the ACMG/AMP variant interpretation guidelines for genetic hearing loss. Hum. Mutat..

[22] Mazzoli M., Van Camp G., Newton V., Giarbini N., Declau F., Parving A. (2003). Recommendations for the description of genetic and audiological data for families with nonsyndromic hereditary hearing impairment. Audiol. Med..

[23] Tamagawa Y., Ishikawa K., Ishikawa K., Ishida T., Kitamura K., Makino S., Tsuru T., Ichimura K. (2002). Phenotype of DFNA11: A nonsyndromic hearing loss caused by a myosin VIIA mutation. Laryngoscope.

[24] Sato O., Komatsu S., Sakai T., Tsukasaki Y., Tanaka R., Mizutani T., Watanabe T.M., Ikebe R., Ikebe M. (2017). Human myosin VIIa is a very slow processive motor protein on various cellular actin structures. J. Biol. Chem..

[25] Azaiez H., Booth K.T., Ephraim S.S., Crone B., Black-Ziegelbein E.A., Marini R.J., Shearer A.E., Sloan-Heggen C.M., Kolbe D., Casavant T. (2018). Genomic Landscape and Mutational Signatures of Deafness-Associated Genes. Am. J. Hum. Genet..

[26] Gan N.S., Oziębło D., Skarżyński H., Ołdak M. (2023). Monogenic Causes of Low-Frequency Non-Syndromic Hearing Loss. Audiol. Neurootol..

[27] Kallman J.C., Phillips J.O., Bramhall N.F., Kelly J.P., Street V.A. (2008). In search of the DFNA11 myosin VIIA low- and mid-frequency auditory genetic modifier. Otol. Neurotol..

[28] Kobayashi M., Miyagawa M., Nishio S.Y., Moteki H., Fujikawa T., Ohyama K., Sakaguchi H., Miyanohara I., Sugaya A., Naito Y. (2018). WFS1 mutation screening in a large series of Japanese hearing loss patients Massively parallel DNA sequencing-based analysis. PLoS ONE.

[29] Kim S.Y., Kim A.R., Kim N.K., Kim M.Y., Jeon E.H., Kim B.J., Han Y.E., Chang M.Y., Park W.Y., Choi B.Y. (2015). Strong founder effect of p. P240L in *CDH23* in Koreans and its significant contribution to severe-to-profound nonsyndromic hearing loss in a Korean pediatric population. J. Trans. Med..

[30] Palombo F., Al-Wardy N., Ruscone G.A., Oppo M., Kindi M.N., Angius A., Al Lamki K., Girotto G., Giangregorio T., Benelli M. (2017). A novel founder *MYO15A* frameshift duplication is the major cause of genetic hearing loss in Oman. J. Hum. Genet..

[31] Ramzan K., Al-Owain M., Al-Numair N.S., Afzal S., Al-Ageel S., Al-Amer S., Al-Baik L., Al-Otaibi G.F., Hashem A., Al-Mashharawi E. (2020). Identification of *TMC1* as a relatively common cause for nonsyndromic hearing loss in the Saudi population. Am. J. Med. Genet. B Neuropsychiatr. Genet..

[32] Matsunaga T., Mutai H., Kunishima S., Namba K., Morimoto N., Shinjo Y., Arimoto Y., Kataoka Y., Shintani T., Morita N. (2012). A prevalent founder mutation and genotype-phenotype correlations of *OTOF* in Japanese patients with auditory neuropathy. Clin. Genet..

[33] Naito T., Nishio S.Y., Iwasa Y., Yano T., Kumakawa K., Abe S., Ishikawa K., Kojima H., Namba A., Oshikawa C. (2013). Comprehensive genetic screening of *KCNQ4* in a large autosomal dominant nonsyndromic hearing loss cohort: Genotype-phenotype correlations and a founder mutation. PLoS ONE.

[34] Maekawa K., Nishio S.Y., Abe S., Goto S.I., Honkura Y., Iwasaki S., Kanda Y., Kobayashi Y., Oka S.I., Okami M. (2019). Mutational spectrum and clinical features of patients with *LOXHD1* variants identified in an 8074 hearing loss patient cohort. Genes.

[35] Yasukawa R., Moteki H., Nishio S.Y., Ishikawa K., Abe S., Honkura Y., Hyogo M., Mihashi R., Ikezono T., Shintani T. (2019). The prevalence and clinical characteristics of *TECTA*-associated autosomal dominant hearing loss. Genes.

[36] Chen S., Francioli L.C., Goodrich J.K., Collins R.L., Kanai M., Wang Q., Alföldi J., Watts N.A., Vittal C., Gauthier L.D. (2024). A genomic mutational constraint map using variation in 76,156 human genomes. Nature.

